# Sympathetic Nerve Activity and Baroreflex are Strongly Altered in a Context of Severe Hypertension Using the Spontaneously Hypertensive Rat Model Associated with Chronic Reduction of Nitric Oxide

**DOI:** 10.1155/2021/4808657

**Published:** 2021-11-25

**Authors:** Christine Vayssettes-Courchay, Jonathan Melka, Clothilde Philouze, Najah Harouki

**Affiliations:** Servier Research Institute, 11 Rue des Moulineaux, Suresnes 92150, France

## Abstract

The aim of our study is to investigate the sympathetic output and baroreflex via renal sympathetic nerve activity (RSNA) recording in a model of severe hypertension which exhibits arterial, cardiac, and renal damages, the spontaneously hypertensive rat (SHR) under lowered NO bioavailability. SHR are treated from 18 to 20 weeks of age with a low dose of L-NAME, a NO synthase inhibitor, in drinking water (SHRLN) and compared to SHR and normotensive Wistar Kyoto (WKY) rats. After the two-week treatment, rats are anesthetized for RSNA, mean blood pressure (MBP), and heart rate (HR) recording. MBP is higher in SHR than in WKY and higher in SHRLN than in SHR. Compared to WKY, SHR displays an alteration in the baroreflex with a displacement of the sympathoinhibition curve to highest pressures; this displacement is greater in SHRLN rats. The bradycardic response is reduced in SHRLN compared to both SHR and WKY. In hypertensive rats, SHR and SHRLN, basal RSNA is modified, the maximal amplitude of burst is reduced, but minimal values are increased, indicating an increased basal RSNA with reduced bursting activity. The temporal correlation between RSNA and HR is preserved in SHR but altered in 10 SHRLN out of 10. The RSNA inhibition triggered by the Bezold–Jarisch reflex activation is not modified in hypertensive rats, SHR or SHRLN, in contrast to that triggered by the baroreflex. Histological analysis of the carotid bifurcation does not reveal any abnormality in SHRLN at the level of the carotid sinus. In conclusion, data indicate that the sympathetic outflow is altered in SHRLN with a strong reduction of the baroreflex sympathoinhibition and suggest that its central pathway is not involved. These additional results on SHRLN also confirm the usefulness of this model of severe hypertension with multiple target organ damages.

## 1. Introduction

Hypertension (HT) is one of the most widespread pathologies worldwide. A number of highly effective drug treatments exist; however, blood pressure control is still not satisfactory, and the prevalence remains very high. HT is known as a major risk factor for the development of cardiovascular diseases, morbidity, and mortality [1]. Moreover, when long-term HT is associated with aging and/or other risk factors such as metabolic diseases, the resulting cardiovascular risk is highly increased. Indeed, HT is severely associated with target organ damages, i.e., the brain, heart, kidneys, arteries, and eyes as listed in Section 6 of the 2020 International Guidelines [2, 3], the sympathetic nervous system being involved at various levels [2, 3]. Current HT animal models, such as the spontaneously hypertensive rat (SHR), do not recapitulate the severe stage of human pathology. The SHR exhibits an early hypertension which increases slowly and is associated with a progressive vascular and cardiac remodeling. When SHR gets older, it shows some similarities with human HT such as impaired renal functions, vascular dysfunction, and stiffness, increased central blood pressure, and ventricular hypertrophy [4]. Therefore, the old SHR is more interesting to mimic human severe hypertension, but this model is difficult to manage, requiring about 12 months to develop hypertension, implying long preclinical studies. We thus developed a model derived from Zhou and Frolich [5]. Adult SHR are treated during 2 weeks with a moderate dose of nonspecific nitric oxide synthase inhibitor L-nitroarginine methyl ester (L-NAME), known to increase hypertension, blood pressure variability, arterial stiffening associated with arterial remodeling and fibrosis, endothelial dysfunction, kidney as well as cardiac dysfunction [6–13]. Kidney alterations include microalbuminuria, proteinuria, glomerular arteriolar constriction, nephropathy, interstitial and vascular fibrosis, and sclerosis. Cardiac alterations include ventricular hypertrophy, myocardial fibrosis and ischemia, impaired cardiac hemodynamics, cardiomyocytes degeneration, and reduced coronary reserve. Altogether, SHR under L-NAME treatment (SHRLN) is a model exhibiting severe HT, similar to that of aged hypertensive patients and aged SHR and associated with target organ damages. We hypothesized that such pathological patterns are likely associated with alterations in the sympathetic control of blood pressure. The aim of the study is to evaluate the basal sympathetic nervous system activity and baroreflex to investigate, respectively, long-term and instantaneous blood pressure control in the SHRLN model.

## 2. Methods

### 2.1. Animals

This study was approved by the Animal Experimentation Ethical Committee of the Servier Research Institute and conforms with the 2010 European Directive for the use of experimental animals and with the National Institutes of Health (NIH) guidelines for the care and use of laboratory animals.

Three groups of 18-week-old male rats are used (from Janvier, Le Genest St Isle, France): normotensive Wistar Kyoto (WKY), SHR, and SHR treated with the NOS inhibitor L-NAME (Sigma) in drinking water at 50 mg/L, leading to a dose of 6 mg/kg/day. Rats' weight and water consumption are controlled every 2 days, and L-NAME is renewed and concentration adjusted. The treatment is maintained during 2 weeks from 18 to 20 weeks of age.

### 2.2. Measurements

At 20 weeks of age, the rats are anesthetized with pentobarbital sodium (50 mg/kg i.p. as the initial dose, followed by a continuous infusion along the experiment, at 12 mg/kg/h through the penis vein). The use of pentobarbital allows to maintain blood pressure rather high and therefore to obtain RSNA recordings with typical rhythms detectable, to maintain a satisfactory level of anesthesia for animal welfare, and to avoid uncontrolled movements at the level of the electrode, only leaving the respiratory movement to be managed. The trachea is cannulated, and artificial ventilation is provided with a Harvard rodent ventilator at a frequency of 70 cycles/min, with a tidal volume of 2.4–2.8 ml during surgery. The rats can then breathe freely. Body temperature is checked by rectal temperature, and the rats laying on a steel plate were maintained constantly at 38°C via controlled warm water flow. Systemic arterial blood pressure is measured from the right common femoral artery via a Statham P10 EZ pressure transducer connected to a pressure recorder (Gould). Heart rate is measured with a Gould cardiotachometer triggered by the pressure pulse. The femoral vein is cannulated for the i.v. administration of drugs.

Left renal nerve is exposed by retroperitoneal dissection. The procedures for RSNA recording are derived from [14]. The nerve is dissected free and placed on a bipolar stainless-steel electrode through a surgical binocular magnifier. The nerve signal is amplified (DAM 60, WPI) with the band-pass filter set at 30 Hz and 1 kHz and is rectified by a Gould integrator.

For the analysis of the sympathetic nerve rhythm, a Spike 2 software (CED) is used, and the rectified signal is recorded in *µ*V/100 ms and cumulated for 60 s by 500 ms periods triggered by the heart rate.

To measure the mean sympathetic renal nerve activity (RNA), the signal is recorded in *µ*V/s and analyzed, together with mean blood pressure (MBP) and heart rate (HR), via the data acquisition and analysis system AcqKnowledge 3.7.3 (Biopac). Absolute values of RNA are corrected by subtracting the residual electrical output. Nerve activity in *µ*V cannot be compared between rats as the value is not only representative of nerve outflow but also of the electrode, nerve isolation, and environment. The changes are thus measured individually in % from the control value. Additionally, we analyze the basal RSNA and bursting activity by averaging the *µ*V/100 ms over a 60 s period. The maximal burst values and the minimal values are reported to the mean RNSA over this period. A 30 min stabilization period is allowed before drug administration.

### 2.3. Reflexes Stimulation

The baroreflex is induced by successive administration of diltiazem (Sigma) at 1 mg/kg into the penis vein, followed by infusion of L-phenylephrine (Sigma) at 20 *µ*g/kg/min into the jugular vein. The baroreflex curve is constructed by plotting the RNA/pressure from the lowest pressure after diltiazem injection to the highest pressure after phenylephrine administration. For each rat, the RNA values are averaged by steps of 5 mmHg; therefore, the values can be averaged in each group of rats (*n* = 9 WKY, *n* = 9 SHR, and *n* = 10 SHRLN).

The Bezold–Jarisch reflex is elicited by a rapid injection of *m-*chlorophenylbiguanide hydrochloride (1-3-20 *µ*g/kg) (CPBG, RBI Biochemicals) via the right jugular vein, close to the heart, in an additional group of rats (*n* = 7 WKY, *n* = 5 SHR, and *n* = 7 SHRLN).

### 2.4. Analysis

At the end of the experiment, the nerve residual electrical background is measured after application of xylocaine 5% on the nerve. The rats are then euthanized by a lethal dose of pentobarbital sodium. In 5 SHR and 5 SHRLN, the common carotid and carotid sinus are removed together and embedded in paraffin. Five longitudinal sections of 5 *µ*m thickness are obtained per artery. Some sections are stained by hemalum-eosin; in others, chromogranin and smooth muscle actin (SMA) are detected by immunochemistry and reveal neural terminals and smooth muscle cells, respectively. The intima-media thickness is measured.

Blood pressure and changes in blood pressure are measured in mmHg, heart rate is measured in beats per min (bpm), and changes in heart rate in % from the basal value. RNA is expressed in %. The data are expressed as means S.E.M. The significance of the difference between dose-response curves is assessed by two-way ANOVA and that between the groups by a one-way ANOVA with Tukey's multiple comparison test. Unpaired Student's *t*-test is used for histological data. In all cases, the difference is considered significant at values of *P* < 0.05.

## 3. Results

### 3.1. Sympathetic Nerve Activity

Basal MBP is higher in SHR than in WKY and even higher in SHRLN after stabilization, and the HR is lower in hypertensive rats. Surgery for RSNA recording lowers MBP and blunts these differences between the 3 groups (Table 1).

**Figure 1:**
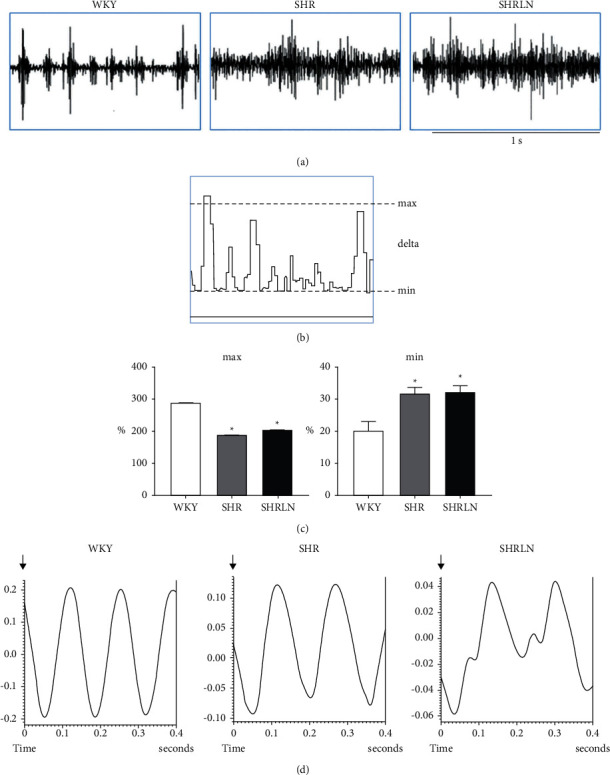
(a) Example of raw recordings of renal sympathetic nerve activity in normotensive WKY and hypertensive SHR and SHRLN. (b) Example of RNA recording for burst analysis, thus rectified and integrated in *µ*V/100 ms. (c) RNA analysis of burst: the maximal value/mean RSNA representing maximal amplitude (max); the minimal value/mean RSNA. ^*∗*^ Significantly different from WKY (one-way ANOVA). (d) Examples of online analysis of the RSNA-HR temporal correlation; RSNA is cumulated over 400 ms periods, triggered by systolic pressure. All examples shown are taken from our present experiments.

Figures 1(a)–1(d)) show typical recording from the experiment described in the present study. Basal RNA is increased in SHR compared to WKY and more in SHRLN as shown in Figure 1(a). Intergroup quantification of RNSA being impossible, we analyze the bursting activity via the RSNA integrated/100 ms. In SHR and SHRLN, we observe a similar reduction of the maximal burst values, thus of burst amplitude, but an increase in the minimal RSNA values which may reveal a global increase in RSNA (Figures 1(b)–1(c)).

The RNA-HR temporal correlation is almost normal in SHR compared to WKY, but this correlation is greatly altered in 10 out of 10 rats in the SHRLN group (Figure 1(d)).

### 3.2. Baroreflex

**Figure 2:**
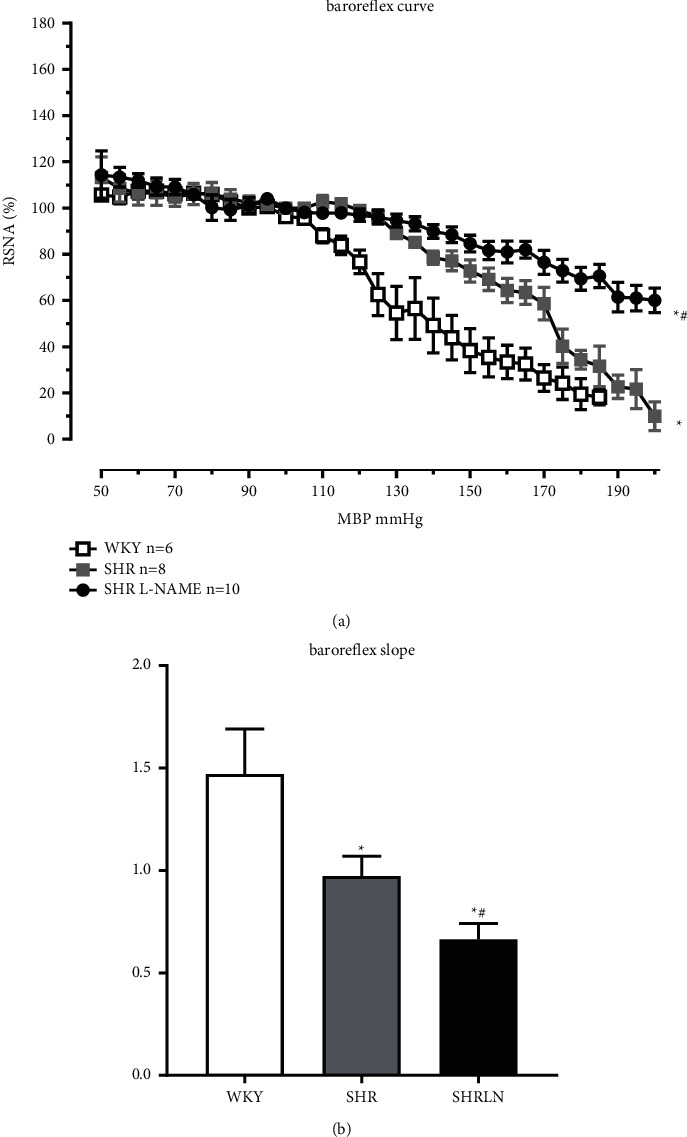
(a) The baroreflex curve in the 3 groups of rats, normotensive WKY and hypertensive SHR and SHRLN. The curves are significantly different between groups and the values differ significantly in function of MBP (two-way ANOVA, *P* < 0.05 for both pressure and groups). (b) The slope of the descending part of the curve (increase in MBP), unpaired Student*'*s *t*-test. ^*∗*^*P* < 0.05 versus WKY. #*P* < 0.05 SHRLN versus SHR.

The baroreflex curve is significantly reduced in SHR compared to WKY, and this reduction is even more marked in SHRLN. This is shown by the RNA/pressure curve of the baroreflex and by the slope of the curves (Figure 2). The MBP plateau value is displaced to higher pressure in SHRLN (135 ± 11 mmHg versus 110 ± 2 in WKY and 113 ± 4 in SHR).

Moreover, the bradycardia induced by L-phenylephrine is significantly reduced in SHRLN (−26 ± 10 bpm) when compared to SHR (−92 ± 21 bpm) and WKY (−107 ± 17 bpm).

Diltiazem induces a more pronounced hypotension in hypertensive than in normotensive rats. However, MPB remains significantly more elevated in SHRLN after diltiazem. After L-phenylephrine, the MBP is higher in hypertensive compared to WKY (Table 1).

### 3.3. Bezold–Jarisch Reflex

In another series of rats, the Bezold–Jarisch reflex is tested with 3 doses of CPBG.

The sympathetic nerve inhibition triggered by CPBG injections is similar in WKY, SHR, and SHRLN at 3 doses (Table 2).

The subsequent hypotension is increased in SHR, not in SHRLN when compared WKY; the subsequent bradycardia in SHR is similar to that measured in WKY and reduced in SHRLN at the highest dose (Table 2).

### 3.4. Histology

**Figure 3:**
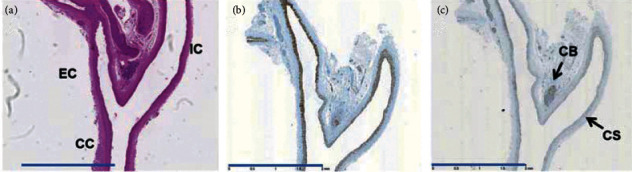
Carotid sinus histology. (a) Hemalum-eosin staining, (b) smooth muscle actin, and (c) chromogranin A. Blue scale bar is 2 mm. There is no difference between SHR and SHRLN. CC, common carotid; EC, external carotid; IC, internal carotid; CB, carotid body; CD, carotid sinus. The examples shown are SHR sections, and no difference was visible on SHRLN sections.

The values measured from histological sections are given in Table 3. The common, internal, and external carotid arteries, as well as the carotid sinus containing baroreceptors and carotid body containing chemoreceptors, are visible on the carotid bifurcation longitudinal sections (Figure 3). No histological difference is noted in SHRLN compared to SHR, with either hemalum-eosin, SMA, or chromogranin A.

The intima/media thickness of the internal carotid, at the level of the carotid sinus, appears thinner on its internal wall than its external wall in SHR. This internal wall is slightly increased in SHRLN without reaching significance, but the ratio internal wall/external is significantly higher in SHRLN than in SHR. The internal and external walls of the external carotid do not differ in SHR and SHRLN.

## 4. Discussion

The antihypertensive treatments acting centrally on the sympathetic outflow are not first-line treatments according to the guidelines of HT management. However, the sympathetic outflow plays a role in the resistant or refractory HT, and therapeutic manipulations have been developed, such as the baroreflex stimulation on the one hand and the renal denervation on another hand. Moreover, HT, in association with other risk factors, remains a crucial determinant in the global cardiovascular risk [2, 3, 15]. Therefore, a new treatment, or a new combination of treatments, needs to be tested on a preclinical rodent model developing a severe HT and target organ damages.

A number of useful HT rat models exist (the old SHR, the specific salt-dependent models as the DOCA-salt and the Dahl salt-sensitive rats, the normal rats treated with high doses of NO synthase inhibitor, the vitamin D-nicotine rat (VDN), and the double transgenic rat (dTGR) model), but we focused our study on the SHRLN, in which L-NAME is used at low dose. We previously characterized SHRLN as a model of severe HT with target organ damages [10, 13, 16], and this model is also of interest to investigate the major role of NO loss in the pathology [17]. Other teams, using a similar model with a different protocol for L-NAME dose and duration, also demonstrated the presence of endothelial, cardiac, and kidney dysfunction in SHRLN [6–9]. Interestingly, despite a major renal dysfunction, this model presents a normal renal endothelial function, the EDHF pathway fully compensating the NO dysfunction [12].

The questions that we addressed were as follows. (1) Is the sympathetic output altered in this model? (2) If yes, is the baroreflex also altered? (3) And if yes, is it possible to define whether the origin is at the afferent, central or efferent pathway level? Our results answer the first two questions and provide a partial response to the last point.

Indeed, our data show an increase in sympathetic activity in SHR and SHRLN. It can be related to the high blood pressure level in this model and to a vicious circle in the development of severe HT, as previously stated [18, 19]. NO induces several feedback controls at peripheral and central levels that are likely reduced in the SHRLN model, as it occurs in aged hypertensive humans. This reduction may be responsible for the increased sympathetic output. The receptors and pathways possibly involved in the observed sympathoactivation are numerous. Afferents signals can be modified due to neurohumoral changes, and the complex central pathway regulating sympathetic tone presents several candidates that could play a role as central receptors. In the rostral ventrolateral medulla, the main central area of sympathetic and blood pressure control, adrenergic and serotoninergic receptors, and several other receptors, as well as angiotensin pathways, could be involved in the sympathoactivation observed in the model.

We observed that in SHR, the temporal correlation between RSNA and HR is preserved. In contrast, the increase in RSNA in SHRLN is associated with an alteration of this RSNA-HR temporal correlation, which strengthens the hypothesis of a sympathetic output contribution in the development of the pathology in this model. An increase in sympathetic activity is not necessarily associated with a loss of the time correlation with HR. Hence, under systemic inflammation, a huge sympathoactivation is observed, but the RSNA/HR correlation remains preserved until the fatal final drop in MBP [14].

The baroreflex is altered in the SHR, as reflected by the increased MBP necessary to reduce RSNA (the curve is displaced to the high pressures), in agreement with previous published data (for example [20, 21]). In SHRLN, the baroreflex alteration is more severe than in SHR, and there is almost no decrease in RSNA when blood pressure increases. These data are important for the model as the baroreflex plays a major role in the control of blood pressure [22].

We tested the Bezold–Jarisch reflex, which also induces a drop in RSNA and is involved in human fall in blood pressure [23, 24]. This cardiopulmonary reflex induces bradycardia and hypotension due to a rapid fall in sympathetic output. The Bezold–Jarisch reflex is due to the activation of cardiac, pulmonary, and bronchial sensory receptors and of receptors located in the carotid body (nodose ganglia). The afferent pathway involves cardiopulmonary vagal fibers to central cardiovascular areas and then involves central medullary areas similar to that of the baroreflex [25, 26] to induce a fall in sympathetic outflow. Thus, if the central areas which control the baroreflex are involved in the sympathoinhibition reduction, the sympathoinhibition induced by the Bezold–Jarisch reflex would be also altered.

In our study, we show that the inhibition of the RNA is not altered during the Bezold–Jarisch reflex, indicating that the central medullary pathways are likely not involved in the reduced RNA inhibition during the baroreflex.

The sympathetic activity inhibition is triggered by the increase in MBP in the baroreflex but triggers the reduction in MBP and HR in the Bezold–Jarisch reflex. Concerning the modifications noted in blood pressure or HR responses, we do not have enough elements to conclude, except that these changes are likely due to peripheral alterations.

A limit of our study is that anesthesia and surgery affect the basal hemodynamic values. In our experimental conditions, the blood pressure level and HT reached in SHRLN are close to that of conscious animals previously studied in telemetry [11]. However, after surgery, basal MBP is reduced to SHR values. The second limit is inherent to RSNA recording [19] that does not allow comparison between groups of animals, except in % of changes from the control value. Thus, the increased basal RSNA in SHRLN compared to SHR is visualized but not reflected by the burst analysis.

The model exhibits also a strong arterial stiffening, due to both increased blood pressure and arterial wall remodeling [10, 11, 13]. The overall knowledge on the SHRLN allows suggestion that the alteration of the baroreflex in this model could be due to the stiffening at the arterial localization of baroreceptors, i.e., the aortic arch and carotid bifurcation.

The histological analysis performed in the present study does not reveal significant differences in neuronal and smooth muscle cells abundance between SHRLN and SHR carotid sinus. Only a slight increase on the internal wall of the internal carotid at the level of the carotid sinus is noticed. Further analyses are needed to test the hypothesis of a role of arterial stiffening, by measuring the arterial wall stiffness composition at the carotid sinus level. The aortic arch vascular wall, another site of baroreceptors, was previously shown to present thickening and fibrosis in this model [11]. The reduction in NO production has been measured in our laboratory [11, 12]; however, an analysis of inducible versus endothelial NO production, both at the aortic arch and carotid sinus, should be performed in a future study.

In conclusion, the model of SHR with reduced levels of nitric oxide presents a strong alteration of the sympathetic activity and of the baroreflex aptitude to reduce blood pressure. The data suggest that this phenomenon likely does not involve the central pathways but a loss of afferent signal.

These sympathetic and baroreflex alterations are additional elements to the previously demonstrated arterial, renal, cardiac, and hemodynamic dysfunctions that confirm the usefulness of the SHRLN model for preclinical studies.

## Figures and Tables

**Table 1:** Basal and baroreflex data.

		WKY, *n* = 9	SHR, *n* = 10	SHRLN, *n* = 10
MBP, mmHg	Basal	151 ± 5	192 ± 4^*∗*^	218 ± 8^*∗*^^#^
	Control	126 ± 3^$^	142 ± 8^$^	156 ± 9^*∗*^^$^
HR, bpm	Basal	443 ± 9	401 ± 8^*∗*^	379 ± 10^*∗*^
	Control	437 ± 6	374 ± 11^*∗*^	355 ± 9^*∗*^
Diltiazem	MBP	49 ± 3	53 ± 3	60 ± 4^*∗*^
	Delta MBP	−77 ± 4	−89 ± 8	−96 ± 8
	HR, bpm	449 ± 12	371 ± 11^*∗*^	329 ± 11^*∗*^
L-phenylephrine	MBP	195 ± 3	226 ± 4^*∗*^	237 ± 10^*∗*^
	Delta MBP	+69 ± 5	+79 ± 8	+81 ± 13
	HR, bpm	336 ± 17	279 ± 21	329 ± 11

2-way ANOVA and Tukey's multiple comparison test, *P* < 0.05. ^*∗*^SHR or SHRLN versus WKY. ^#^SHRLN versus SHR. Student's *t*-test: ^$^*P* < 0.05 control value versus basal. Basal values of mean blood pressure (MBP) and heart rate (HR) after stabilization (before surgery) and control values after surgery. Effect of diltiazem (1 mg/kg) and L-phenylephrine (20 *µ*g/kg/min), i.v.

**Table 2:** Bezold–Jarisch reflex data.

		Basal	CPBG 1 *µ*g/kg	CPBG 3 *µ*g/kg	CPBG 20 *µ*g/kg
RSNA, %	WKY *n* = 7	—	−58 ± 5	−65 ± 5	−67 ± 5
	SHR *n* = 5	—	−70 ± 6	−76 ± 4	−73 ± 6
	SHRLN *n* = 7	—	−67 ± 10	−78 ± 4	−81 ± 4

MBP, mmHg	WKY	113 ± 4	−19 ± 4	−30 ± 7	−49 ± 4
	SHR	176 ± 8^*∗*^	−46 ± 4^*∗*^	−72 ± 7^*∗*^	−80 ± 8^*∗*^
	SHRLN	168 ± 14^*∗*^	−32 ± 6	−43 ± 7^#^	−50 ± 9^#^

HR, bpm	WKY	408 ± 15	−49 ± 10	−107 ± 15	−257 ± 32
	SHR	357 ± 20	−108 ± 35	−179 ± 32	−257 ± 17
	SHRLN	354 ± 12	−57 ± 16	−116 ± 31	−142 ± 34^#^

2-way ANOVA and Tukey's multiple comparison test, *P* < 0.05. ^*∗*^SHR or SHRLN versus WKY. ^#^SHRLN versus SHR.

**Table 3:** Carotid sinus histological data.

Intima/media thickness		SHR, *n* = 5	SHRLN, *n* = 5
Internal carotid	Internal wall	48 ± 2	59 ± 6
	External wall	75 ± 3	73 ± 4
	Ratio i/e	0.66 ± 0.04	0.84 ± 0.02^#^
External carotid	Internal wall	66 ± 1	64 ± 1
	External wall	46 ± 3	77 ± 2
	Ratio i/e	0.87 ± 0.03	0.83 ± 0.03

Unpaired Student's *t*-test. *P* < 0.05. ^#^SHRLN versus SHR.

## Data Availability

All data generated or analyzed during this study are included in this article.
